# Interpretation of regulatory factors for 3D printing at hospitals and medical centers, or at the point of care

**DOI:** 10.1186/s41205-022-00134-y

**Published:** 2022-02-01

**Authors:** Brian G. Beitler, Paul F. Abraham, Alyssa R. Glennon, Steven M. Tommasini, Lisa L. Lattanza, Jonathan M. Morris, Daniel H. Wiznia

**Affiliations:** 1 Department of Orthopaedics and Rehabilitation, Yale School of Medicine, 800 Howard Ave 1st Floor, CT 06519 New Haven, USA; 2 Department of Orthopaedic Surgery, Keck School of Medicine of USC, Los Angeles, CA USA; 3 Materialise, Plymouth, MI USA; 4 Department of Biomedical Engineering, Yale University, New Haven, CT USA; 5 Department of Radiology, The Mayo Clinic, Rochester, MN USA; 6 Department of Mechanical Engineering & Materials Science, School of Engineering & Applied Science, Yale University, New Haven, CT USA

**Keywords:** 3D Printing, Orthopedics, FDA, Point-of-care (PoC) 3D Printing, Medical device design

## Abstract

3D printing is revolutionizing the medical device landscape through its ability to rapidly create patient-specific anatomic models, surgical instruments, and implants. Recent advances in 3D printing technology have allowed for the creation of point-of-care (PoC) 3D printing centers. These PoC centers blur the line between healthcare provider, medical center, and device manufacturer, creating regulatory ambiguity. The United States Food & Drug Administration (FDA) currently regulates 3D printed devices through existing medical device regulations. However, the FDA is increasingly interested in developing guidelines and regulations specifically for PoC 3D printing due to its rapid adoption across the healthcare institutions. In this article, we review the regulatory framework that governs medical devices, discuss how PoC 3D printing falls within this framework, and describe a novel conceptual framework that the FDA has proposed. Finally, through analysis of the aforementioned regulations and discussions with industry medical 3D printing regulatory experts, we provide recommendations for PoC medical 3D printing best practices so that institutions are best positioned to utilize this revolutionary technology safely and effectively.

## Background

3D printing is a process involving the creation of a physical object from a digital 3D model through the method of additive manufacturing. Its use in medicine has been expanding over the years and its applications are numerous. These applications include facilitating surgical planning through the use of patient-specific anatomic models [1], manufacturing patient-specific instrumentation (PSI) including cutting guides and jigs [2], and manufacturing implants such as spine cages [3] as well as arthroplasty components [4]. When manufactured and sold by medical device companies, the United States Food & Drug Administration (FDA) has regulated these devices through existing medical device regulations [5]. Furthermore, the FDA’s Additive Manufacturing Working Group held a workshop in October 2014 entitled “Additive Manufacturing of Medical Devices: An Interactive Discussion on the Technical Considerations of 3D Printing” to learn from stakeholders about the technical challenges of medical device 3D printing and best practices for process and product validation [5]. The feedback from this workshop served as the basis for a guidance document entitled “Technical Considerations for Additive Manufactured Medical Devices” [6] issued in December 2017, which provided 3D printing manufacturers with guidance for device design, manufacturing, and testing.

With recent advances in 3D printed medical device research, there has been a rapid increase in point-of-care (PoC) manufacturing [7, 8]. We acknowledge that there is no universally accepted definition of PoC 3D printing. We define PoC 3D Printing as the just-in-time creation of 3D printed diagnostic use anatomic models, surgical instruments, or other medical devices based on a patient’s medical imaging data, either at the place of patient care (such as a hospital) or in a centralized facility owned by the health care organization [7]. We recognize that this definition does not include 3D printing beyond patient-matched devices. In short, our definition of PoC 3D printing is when a healthcare provider (or healthcare organization), not a medical device manufacturer, creates a patient specific 3D printed medical device. While PoC 3D printing has the potential to improve patient outcomes and reduce operative time and costs, such a design and manufacturing setting blurs the line between health care provider and device manufacturer and creates regulatory ambiguity. Individual medical practitioners using 3D printed devices in a PoC fashion under the practice of medicine are not subject to Premarket Notification or Premarket Approval pathways, as long as they are not marketing or selling these devices [9]. Currently the FDA remains undecided about the best way to regulate PoC printing centers, though they have recently begun working with stakeholders to develop a regulatory framework [9]. The purpose of this review is to consolidate currently available information pertaining to the regulation of PoC 3D printed devices and to summarize the most current proposed regulatory framework for PoC printing of medical devices.

## Methods

Embase and Internet sources were screened for official FDA documents, regulations, and editorials pertaining to 3D printing and/or additive manufacturing. In order to restrict internet sources to official FDA documents and regulations, Google was searched for the following terms: site:.gov AND ((3D OR 3-D OR three-dimensional OR additive) AND (printing OR manufacturing) AND (medical OR surgical OR clinical OR diagnostic OR treatment OR orthopedic)).

Since the FDA has not yet given specific guidelines concerning PoC 3D printing of medical devices by healthcare institutions or individual medical practitioners [6], we consulted stakeholders and report their varying opinion with the material culled from the literature. Some of these stakeholders are manuscript authors. For example, one author (JMM) was the past-Chair of the Radiological Society of North America (RSNA) 3D Printing Special Interest Group (RSNA 3D SIG). RSNA 3D SIG is the largest collection of physicians, engineers, and industry representatives, who have organized under a national medical organization (RSNA) to address standards and appropriateness criteria for PoC 3D printing of medical devices. Our discussants included experts in PoC 3D printing at the Mayo Clinic, Boston Children’s Hospital, University of Pittsburgh Medical Center, and Stanford University. We also consulted FDA regulatory specialists from industry partners at FormLabs (Somerville, Massachusetts, USA), Zimmer-Biomet (Warsaw, Indiana, USA), Smith & Nephew (Andover, Massachusetts, USA), DePuy Synthes (West Chester, Pennsylvania, USA), Conformis (Billerica, Massachusetts, USA), Axial3D (Belfast, United Kingdom), and Materialise (Leuven, Belgium). The individual consultants appear in the Acknowledgments. Thus, the interpretations described below reflect the opinions of the authors as influenced by the consultant discussions as well as the peer-reviewed and non-peer reviewed literature as cited. Since this review reflects an interpretation, it should be considered in light of the many varying opinions and interpretations for the regulatory and quality milieu for 3D printing in hospitals and medical centers.

## Review

### Approval departments

While the majority of 3D printed devices are regulated through the FDA’s Center for Devices and Radiological Health (CDRH) [10], the FDA’s Center for Biologics Evaluation and Research (CBER) regulates all biological, cellular, or tissue-based applications of additive manufacturing, the FDA’s Center for Drug Evaluation and Research (CDER) regulates all drug applications of additive manufacturing, and the Office of Combination Products (OCP) regulates products that have components that would normally be regulated by different FDA centers.

One point of frequent misunderstanding which the FDA has clarified in past workshops and documents [5, 6] is that the FDA does not regulate use of raw 3D printing materials, final printing material, or specific printing processes for unspecified uses. Rather, it clears specific devices that may or may not be designed through 3D printing (using existing or novel raw materials) for specific clinical indications. However, in Premarket Notification submissions, it may still be useful to include the names of previously cleared devices that share the same additive manufacturing process to help make the case for substantial equivalence [5].

### FDA device classifications

The FDA classifies all medical devices based on the level of regulation necessary to ensure safety and efficacy. Among other things, this classification determines the type and degree of premarket submission necessary before the FDA clears the device to be marketed [11]. Class I devices, which present minimal risk of harm to the user, are typically exempt from Premarket Notification, though like all medical devices they still must follow a set of FDA provisions called General Controls which allow the FDA to ensure the safety of all medical devices [12]. Class II devices—which account for the majority of medical devices and pose greater risk to the user than Class I devices—generally require Premarket Notification (PMN) known as a 510(k). A 510(k) is a premarket submission to demonstrate that a device is substantially equivalent to a legally marketed device (and thus is as safe and effective). Most 3D printed medical devices cleared by the FDA to date have been cleared through this pathway [5]. Devices that do not have a “substantial equivalent” but for which there exists a reasonable assurance of safety (based on specific FDA guidelines), can be classified as Class I or Class II devices through the “de novo” pathway. These devices can be marketed and serve as predicate devices for future 510(k) submissions [13]. Lastly, Class III devices—a minority of devices, which lack equivalents on the market and that sustain or support life, are implanted, or pose relatively high risk of illness or injury—require the more stringent Premarket Approval (PMA) and are not eligible for Premarket Notification through the 510(k) pathway. For most Class III devices, the FDA requires clinical trial evidence of the device’s safety and efficacy as part of PMA requirements.

Most clinical applications for 3D printed devices which could be produced in the PoC setting fall into three categories: diagnostic use anatomic models [14], patient-specific surgical instruments [5], and patient-specific implants [5, 6]. Of these, diagnostic use anatomic models are the most commonly produced in the PoC setting [8]. Anatomic models are typically derived from patients’ computed tomography (CT) imaging, allowing for visualization of patient-specific anatomy in three dimensions [15, 16]. Diagnostic use anatomic models sold by a manufacturer to a hospital typically fall under FDA Class II regulations. In August 2017 the RSNA SIG and the FDA convened a joint session at the FDA White Oak Campus (Silver Spring, MD) to define the current regulatory landscape regarding PoC diagnostic use anatomic models [14]. At that meeting, diagnostic use for a 3D printed anatomic model was defined. All United States companies that currently manufacture and sell patient specific 3D printed anatomic models to physicians and to hospitals are required to follow the guidelines defined at that meeting jointly held by the RSNA and the FDA. Before the meeting jointly held by the RSNA and the FDA [14], there was suggestion in the literature that could be interpreted so that PoC patient matched anatomic models could have been considered medical image hardcopies and not medical devices at all [5, 17]. However, this is now known to not be the case. In this argument that is no longer valid, 3D printers would be considered medical image hardcopy devices (21 CFR 892.2040 Medical Image Hardcopy Device).

Patient-specific surgical instruments and patient-specific implants typically also fall under Class II regulations, but may require Class III regulations if they pose unique safety or efficacy considerations or lack a substantially equivalent predicate device [5]. Though currently rarer in the PoC setting than diagnostic use anatomic models, these devices also face regulatory uncertainty.

### Quality systems regulations

Legally marketed 3D printed devices are subject to the same regulatory requirements to which similar devices created without 3D printing are subject [6], including Quality Systems (QS) regulations. The purpose of this type of regulation is to ensure that medical devices consistently meet necessary requirements and specifications. Though it remains unclear exactly how they are to be regulated, it is still important that PoC centers manufacture safe devices. Outlined below are some QS regulations that medical device manufacturers must adhere to, and that PoC 3D printing centers should be particularly mindful of when creating their own “best practices.”

1. Monitoring, maintenance protocols, and control of process parameters [6]: Parameters of the 3D printer system being used, such as calibration and maintenance protocols and environmental conditions, must be documented. If the device designer includes interactive steps in the patient matching workflow (e.g. outside transfer of patient data to create the 3D model), they must implement the FDA’s Guidance on the “Content of Premarket Submissions for Management of Cybersecurity in Medical Devices” [18].
2. Imaging: Imaging upon which patient-specific 3D-devices are modeled should be of sufficient resolution to properly capture the areas of interest. In general, the smallest anatomy of interest should be captured on at least 3 sequential DICOM images of a particular series. For small areas of interest, this may dictate the slice thickness of the computed tomography (CT) or magnetic resonance imaging (MRI) images [6, 19].
3. Physical device manufacturing [6].

- Materials [6]: To ensure consistent starting material, for each raw material used the following must be documented: (1) chemical name, common name, trade name, Chemical Abstracts Service number, or recognized consensus material standard; (2) supplier; and (3) material certificates of analysis (COAs) and the test methods used for the COAs. The same should be documented for all additives, processing aids, and cross-linkers used. If the device manufacturer desires to change a material, the effect on the build process and final device safety and efficacy should be investigated and documented. Because reused material (e.g. unsintered powder in powdered bed fusion or uncured resin in stereolithography) can be altered from the starting state, it is important to document the material reuse process and show that it does not negatively affect the final device performance [6].
- Support material [6]: It is common to include support structures such as struts in the manufacturing of a 3D printed device to allow for the printing of complex 3D builds. Common structures requiring support include overhangs, high aspect ratio features that protrude from the main body of the device, internal channels, and thin features prone to distortion. These supports are typically removed through a physical or chemical process after the build is complete. It is vital to detail when supportive materials will be used, how they will be removed, and to explain how the final product is not negatively impacted by support removal.
- Layering and Meshing [6, 19]: Layer thickness of the 3D printed device should be optimized for its intended use. Similar to the imaging selection stage, layer thickness should be determined such that the smallest area of interest is captured on at least three consecutive layers of the 3D printed device. 3D model files are frequently composed of a mesh of triangular faces (the .stl file format), and the number and size of these faces can affect model accuracy. The rationale behind the selected size and number of these triangles in the mesh should be documented.
- Build paths: The build path used to print a 3D printed device must be evaluated and documented. For example, if the delivery system sweeps from left to right on one pass and then right to left on the next pass, the left side of the device has more time to cool and/or harden than the right side, potentially resulting in malorientation or distortion of the device. Details about the build path, such as this, should be documented and potential consequences evaluated. Additionally, the fill density of the device should be specified in parts or components that are not fully dense (i.e., non-solid). If non-solid fill density is used, whether internal voids are externally accessible or externally sealed should be documented. If these internal voids are sealed but may be violated during use, it is important to report the material or gas that fills the voids and the risks associated with patient exposure to these materials or gasses.
- Post-processing [6]: Post-processing steps should not affect the intended use or accuracy of the device, but should only enhance its utility. All post-processing steps should be documented, and a discussion of the effects of post-processing on the materials of the device should be included. For example, heat treatment with hot isostatic pressing (HIP] is used to reduce residual porosity and increase fatigue life in metal 3D printed implants, but has also been shown to reduce the strength of the material [6]. Thus, the potential impact of HIP should be considered in the intended application for the implant.
- Sterilization [20]: Devices intended for use in the operative setting must also adhere to International Organization for Standardization (ISO) standards for sterility and biocompatibility. The sterilization process of additively manufactured devices may change the geometry of the printing material or cause crosslinking of this material, thereby affecting the shape and strength of the 3D printed device. QS regulations should, therefore, document sterility processes, and validation studies must be done to ensure that these sterility processes are compatible with the properties of the materials used in the 3D printed device.
- Biocompatibility [20]: If toxic chemical additives are used to create the 3D printed device, additional testing may be necessary. Common toxic additives include catalysts, binding and curing agents, uncured monomers, and plasticizers. In addition to assuring biocompatibility of these raw materials, manufactures must also assure biocompatibility of the final sterilized product. Furthermore, QS regulations documentation should include biocompatibility validation studies for patient-specific cutting guides (which may deposit debris onto the operative field when used with power tools) and patient-specific implants.

### Labeling

Each 3D printed device should be labeled with a patient identifier, the intended use of the device, the device’s design iteration number, and an expiration date [6]. This labeling can be directly marked on the device itself or documented on the device’s accompanying packaging and labeling. The expiration date for patient-specific devices may be determined based on the patient’s date of imaging rather than the standard process of determining shelf life as patient anatomy may be subject to change over time (e.g., pediatric or oncologic cases). Furthermore, it is possible that the patient may have experienced events, such as trauma, since undergoing the imaging used to model the 3D printed device that may impact the device’s effectiveness or utility. Therefore, the FDA recommends including a precaution in the labeling that the patient should be examined for anatomic changes prior to use of the patient-specific device [6].

### PoC best practices

Although the FDA has not published specific regulations for these PoC printing centers, there have begun discussions with pertinent stakeholders (including engineers, the medical device industry, SME, the ASME, RSNA 3D SIG, PoC manufacturing centers, physicians, and surgeons) such as via a webinar series hosted by the American Society of Mechanical Engineers in 2020 [9]. The FDA has presented a tentative framework for how it might classify and regulate groups that manufacture PoC 3D printed devices in the future [9]. The original framework included Scenarios A-F (to include F as “Other”). Table 1 represents the authors’ interpretation of five scenarios.

**Table 1:** Proposed FDA Framework for Medical PoC 3D Printing

Scenario	Additional Details and Considerations
A healthcare facility prints and uses 3D-printed devices in a way that presents minimal risk in terms of patient safety and ability to print	• Should employ monitoring and risk mitigations strategies • Should leverage existing standards, certifications • Not intended for implants, life-supporting / life‐ sustaining devices or devices that present a serious health risk to patients
A device is designed by manufacturer to be printed by the healthcare facility where the post-processing steps are automatic or self‐contained	• Uses a validated process that has been evaluated by FDA • Device use should be consistent with cleared indications and manufacturer instructions for use
A device is designed by manufacturer to be printed by the healthcare facility where there is more complex manufacturing and post-printing processes	• The healthcare facility would have to have appropriately trained personnel and proper equipment • May require labeling, training, instructions for calibration, or testing on-site in order to facilitate appropriate 3D printing by the healthcare facility
A manufacturer or contract manufacturer is co- located at the healthcare facility	• The healthcare facility doesn’t intend to set up and manage their own 3D facility or the devices are not minimal risk • The healthcare facility is performed by a traditional manufacturer, contract manufacturer, or other 3rd party using their own equipment and personnel
A healthcare facility chooses to become a manufacturer (develop, test, print) and the devices are not minimal risk	• The healthcare facility desires to design and control their own 3D printing operations and the devices are not minimal risk • The healthcare facility is responsible for development/design, testing, and printing • The healthcare facility is responsible for all regulatory requirements
Adapted from the presentation “3D Printing Medical Devices at Point of Care” [9]	

**Figure 1:**
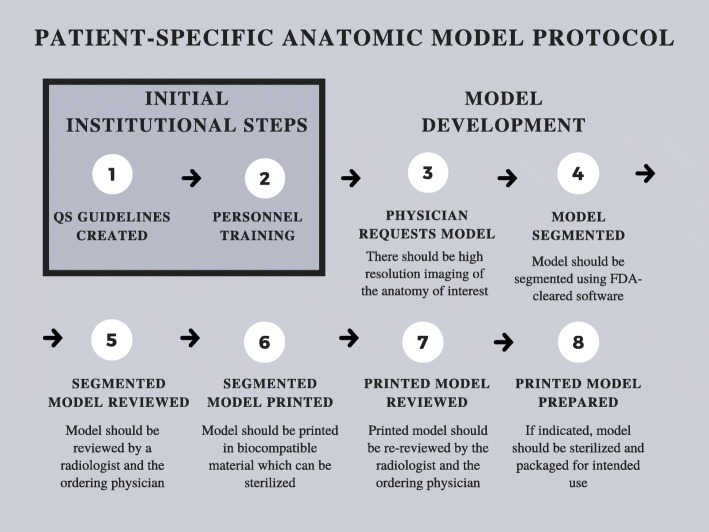
**Fig. 1** Patient-specific anatomic model protocol
- When making 3D printed patient-specific diagnostic use anatomic models at the PoC, it is advised to use FDA-cleared and locally-validated segmentation and Computer-aided Design (CAD) software. Given that each patient-matched medical device will have design constraints based on desired end-part properties and FDA class, the CAD software should be FDA cleared for the appropriate indications.
- To ensure accurate segmentation when 3D printing patient-specific diagnostic use anatomic models, cutting guides, medical implants, or other class I-III devices, a Radiologist and the physician ordering the device should review and approve the segmentation. Training process and qualifications for all segmentation personnel, which may include radiology technologists, industrial designers, or other technologists, should be documented and stored. For oncologic cases, the margins of the tumor should be segmented by a physician with training in disease-specific oncologic imaging.
- It is vital to design validated processes for internal regulation of device manufacturing. For instance, it is important to have an install qualification (IQ), operating qualification (OQ), and performance qualification (PQ) with specific frequencies of phantoms, test prints, inspections, and auditable documentation. Training for all personnel working with the printers should be documented to assure that they can operate them and are up to date.
- Any device used in the sterile field that may have any contact with the patient must undergo biocompatibility testing as outlined in ISO 10,993. As validation studies for sterility and biocompatibility can be time-consuming, costly, and technically challenging, it is advised to outsource these studies to an external laboratory, unless the home institution has an accredited facility.
- PoC 3D printing centers should have conversations with their institutions’ legal teams to discuss risks of PoC designing and manufacturing when planning new applications of PoC 3D printing that may be considered high risk. Furthermore, it should be noted that any application where the hospital will be manufacturing a device that was previously bought outside of the hospital will increase the liability of the hospital as the legal manufacturer.

As the FDA is currently working closely with stakeholders to modify this framework and recommendations, they have not yet been implemented, but specific FDA regulations for PoC 3D printed devices are likely to emerge in the near future [9]. However, in the absence of definitive guidelines for PoC 3D printing centers, we have summarized below some PoC 3D printing “best practices” that we collected by interviewing PoC manufacturing experts:

- Internal regulation is paramount to a successful PoC 3D printing center [21]. For this reason, despite not being subject to Premarket Notification or Premarket Approval pathways, PoC 3D printing centers should create a QS regulations document to detail training documents and processes as well as process parameters and maintenance protocols. QS regulations documents will likely be required by the FDA for certain PoC 3D printing activities when specific PoC 3D printed medical device regulations ultimately emerge. Until then, QS regulations should be equivalent to standards established by the FDA under 21CFR820. Furthermore, it is essential to adhere to standardized protocols for creating 3D printed patient specific devices such as the one outlined in Fig. 1.

## Conclusions

The rapid advancement of 3D printing has made the technology more accessible, but the regulatory requirements are not well defined. The current regulatory landscape is flexible enough to allow for many applications of PoC 3D, as described above. It is anticipated that in the near future the FDA will adopt new regulations which directly address PoC 3D printing, likely based on the conceptual framework developed by the FDA and pertinent stakeholders. To reiterate: the interpretations described above are not statements of fact but reflect the opinions of the authors as influenced by the consultant discussions as well as the peer-reviewed and non-peer reviewed literature as cited. In the meantime, for any institutions establishing PoC 3D printing centers, there are a multitude of resources available to provide guidance, as covered in this review. Furthermore, pending official FDA regulatory guidelines, the CDRH Division of Industry and Consumer Education continues to be an invaluable resource for clarification of the technical topics briefly summarized in this review [5]. Ultimately, while 3D printing promises to continue to revolutionize the practice of medicine, it is important that we continue to move forward safely.

Note: Since this paper was submitted, the FDA has released a discussion paper titled “Discussion Paper: 3D Printing Medical Devices at the Point of Care [22].” The views expressed in this review paper should be understood to have been prepared prior to the release of the FDA discussion paper.

## Data Availability

The datasets used and analyzed during the current study are available from the corresponding author on reasonable request.

## Abbreviations

PoC: Point-of-Care
PSI: Patient-Specific Instrumentation
3D: Three-dimensional
FDA: United States Food & Drug Administration
ASME: American Society of Mechanical Engineers
RSNA 3D SIG: Radiological Society of North America 3D Printing Special Interest Group
CDRH: Center for Devices and Radiological Health
CBER: Center for Biologics Evaluation and Research
PMN: FDA’s Center for Drug Evaluation and Research, Premarket Notification
PMA: Premarket Approval
CT: Computed Tomography
QS: Quality Systems
MRI: Magnetic Resonance Imaging
COAs: Certificates of Analysis
HIP: Hot Isostatic Pressing
ISO: International Organization for Standardization
FFD&C Act: Federal Food, Drug and Cosmetic Act
CAD: Computer-aided Design
IQ: Install Qualification
OQ: Operating Qualification
PQ: Performance Qualification

## References

[1] Bagaria V, Chaudhary K (2017). A paradigm shift in surgical planning and simulation using 3Dgraphy: Experience of first 50 surgeries done using 3D-printed biomodels. Injury.

[2] Chi-Kay L, King-him C, Kin-bong L, Wilson L (2018). Computer-Assisted Planning and Three-Dimensional-Printed Patient-Specific Instrumental Guide for Corrective Osteotomy in Post-Traumatic Femur Deformity: A Case Report and Literature Review. J Orthop Trauma Rehabil.

[3] Lin CY, Wirtz T, LaMarca F, Hollister SJ (2007). Structural and mechanical evaluations of a topology optimized titanium interbody fusion cage fabricated by selective laser melting process. J Biomed Mater Res A.

[4] Koeck FX, Beckmann J, Luring C, Rath B, Grifka J, Basad E (2011). Evaluation of implant position and knee alignment after patient-specific unicompartmental knee arthroplasty. Knee.

[5] Di Prima M, Coburn J, Hwang D, Kelly J, Khairuzzaman A, Ricles L (2016). Additively manufactured medical products - the FDA perspective. 3D Print Med.

[6] Technical Considerations for Additive Manufactured Medical Devices. https://www.fda.gov/media/97633/download. Accessed 14 December 2021.

[7] Arce K, Morris JM, Alexander AE, Ettinger KS (2020). Developing a Point-of-Care Manufacturing Program for Craniomaxillofacial Surgery. Atlas Oral Maxillofac Surg Clin North Am.

[8] Medical Manufacturing Innovations. Physicians as Manufacturers. https://www.sme.org/globalassets/sme.org/media/white-papers-and-reports/3d_printing_fuels_the_rise.pdf. Accessed 14 December 2021.

[9] American Society of Mechanical Engineers (ASME). 3D Printing at the Point of Care. https://resources.asme.org/poc3dp-events. Accessed 14 December 2021.

[10] 3D Printing in Drug Development & Emerging Health Care. https://www.fda.gov/media/125479/download. Accessed 14 December 2021.

[11] Classify Your Medical Device. https://www.fda.gov/medical-devices/overview-device-regulation/classify-your-medical-device. Accessed 14 December 2021.

[12] General Controls for Medical Devices. https://www.fda.gov/medical-devices/regulatory-controls/general-controls-medical-devices#QSR. Accessed 14 December 2021.

[13] De Novo Classification Request. https://www.fda.gov/medical-devices/premarket-submissions/de-novo-classification-request. Accessed 26 December 2021.

[14] FDA/CDRH–RSNA SIG Joint Meeting on 3D Printed Patient-Specific Anatomic Models, August 31. 2017. https://wayback.archive-it.org/7993/20201222130156/https://www.fda.gov/medical-devices/workshops-conferences-medical-devices/fdacdrh-rsna-sig-joint-meeting-3d-printed-patient-specific-anatomic-models-august-31-2017. Accessed 14 December 2021.

[15] Mitsouras D, Liacouras P, Imanzadeh A, Giannopoulos AA, Cai T, Kumamaru KK, George E, Wake N, Caterson EJ, Pomahac B, Ho VB, Grant GT, Rybicki FJ (2015). Medical 3D Printing for the Radiologist. Radiographics.

[16] Mitsouras D, Liacouras PC, Wake N, Rybicki FJ (2020). RadioGraphics Update: Medical 3D Printing for the Radiologist. Radiographics.

[17] Christensen A, Rybicki FJ (2017). Maintaining safety and efficacy for 3D printing in medicine. 3D Print Med.

[18] Content of Premarket Submissions for Management of Cybersecurity in Medical Devices. https://www.fda.gov/regulatory-information/search-fda-guidance-documents/content-premarket-submissions-management-cybersecurity-medical-devices-0. Accessed 14 December 2021.

[19] Chepelev L, Wake N, Ryan J, Althobaity W, Gupta A, Arribas E (2018). Radiological Society of North America (RSNA) 3D printing Special Interest Group (SIG): guidelines for medical 3D printing and appropriateness for clinical scenarios. 3D Print Med.

[20] Use of International Standard ISO 10993-1. “Biological evaluation of medical devices - Part 1: Evaluation and testing within a risk management process.“ https://www.fda.gov/regulatory-information/search-fda-guidance-documents/use-international-standard-iso-10993-1-biological-evaluation-medical-devices-part-1-evaluation-and. Accessed 14 December 2021.

[21] Schulze M, Gosheger G, Bockholt S, De Vaal M, Budny T, Tönnemann M, Pützler J, Bövingloh AS, Rischen R, Hofbauer V (2021). Complex Bone Tumors of the Trunk—The Role of 3D Printing and Navigation in Tumor Orthopedics: A Case Series and Review of the Literature. J Pers Med.

[22] Discussion Paper. 3D Printing Medical Devices at the Point of Care. https://www.fda.gov/medical-devices/3d-printing-medical-devices/3d-printing-medical-devices-point-care-discussion-paper. Accessed 17 January 2022.

